# The race to disasters - is the international relief community ready for future disasters?

**DOI:** 10.1186/s13584-024-00657-1

**Published:** 2024-12-17

**Authors:** Kobi Peleg, Moran Bodas

**Affiliations:** Department of Emergency & Disaster Management, School of Public Health, Faculty of Medical and Health Sciences, Tel-Aviv University, Chaim Levanon 55, Tel-Aviv-Yafo, 6997801 Israel

**Keywords:** International relief, Humanitarian aid, Disasters, Coordination, Paradigm shift

## Abstract

**Background:**

Climate-related disasters have tripled in the past 30 years. Between 2006 and 2016, the global sea levels rose 2.5 times faster than the entire 20th century. More than 20 million people a year are forced out of their homes because of climate change impacts. Rapid urbanization and increasing population density in coastal mega-metropolitan areas will inevitably lead to more large-scale disasters due to extreme weather events, i.e., stronger storms and massive flooding. Despite the inevitability of these events, disaster risk reduction is still locally based in each country, many of which have scarce resources to devote to the activity. It is widely assumed that the global community will respond when a calamity occurs. This perspective article explores the appropriateness of the current international relief and aid paradigm in light of near and middle-term trends in global disasters.

**Main body:**

After briefly summarizing the anticipated effects of global climate change, population growth, and progressive urbanization in low-lying coastal and riverine environments on the frequency and scale of future disasters, this paper examines how existing concepts of international relief following disasters are insufficient to address the challenges to come. Current paradigms are tested against selected case studies demonstrating the growing frequency of mega-disasters. For example, in 2010, the world saw a catastrophic earthquake in Haiti, very large-scale floods in Pakistan, a major earthquake in Chile, and heat waves that resulted in the death of tens of thousands of people in Russia and many more in other countries. However, the world exhausted most of its humanitarian aid, responding to Haiti in January of that year. The review closes with a proposition for a new paradigm to re-organize international relief to meet the challenge posed by our rapidly changing world – one that is more adaptable to the current challenges of climate change and other trends that will almost certainly increase the frequency and intensity of disasters.

**Conclusion:**

The future of international disaster aid depends on our ability to foster greater cooperation between the various organizations and donor countries and more seamless cooperation between both groups and the affected countries or regions. Planning and relief operations should utilize new technologies and innovative financing where feasible. A holistic approach that focuses on building large-scale agreements and coordination mechanisms, teaching citizens how to help each other until aid arrives, and strengthening resilience at the local level will equip communities for adaptive action during a disaster, improve coping and long-term rehabilitation, will lead to a more efficient, fairer and more durable global aid system.

## Background

### The nature of the threat

Over the last 30 years, the number of climate-related disasters has tripled. Between 2006 and 2016, global sea levels rose 2.5 times faster than the entire 20th century [1, 2]. The economic impact of disasters is profound. The secondary insurance company Swiss-Re stated that in 2016 alone, disasters caused damages of 175 billion dollars and 110,000 deaths. Less than a third of these costs (54 billion dollars) were insured [3].

Every year, world leaders and renowned businessmen gather in Davos for the World Economic Forum to discuss the significant risks that will affect the world in the coming years. Every year, a special report is presented at the beginning of the conference, presenting a risk report of the world compiled by hundreds of experts from various fields. The 2019 World Economic Forum report, issued shortly before the emergence of the COVID-19 global pandemic and Russia’s invasion of Ukraine, identified ten risks the world would face in the coming years [4]. Of note, six of these risks are directly related to global climate change, urbanization, and population growth. In particular, extreme weather events was in first place, failure to find solutions and adopt policies for climate change in second place, and major natural disasters such as earthquakes, tsunamis, volcanic eruptions, and huge storms in third place [4]. It is, therefore, understandable that Moomi Mitsutori, the UN special representative for Disaster Risk Reduction, declared: *“This is a climate emergency. The number of disasters resulting from weather or climate has more than doubled over the last 40 years”* [5].

However, the world’s approach to mitigating the risks and consequences of disasters is still based on decisions made by individual countries, which are often constrained by a lack of resources or competing priorities. As a result, effective countermeasures, such as enacting and enforcing appropriate building codes and restricting residential construction and informal settlements in vulnerable flood zones, are often ignored [6, 7].

**Fig. 1:**
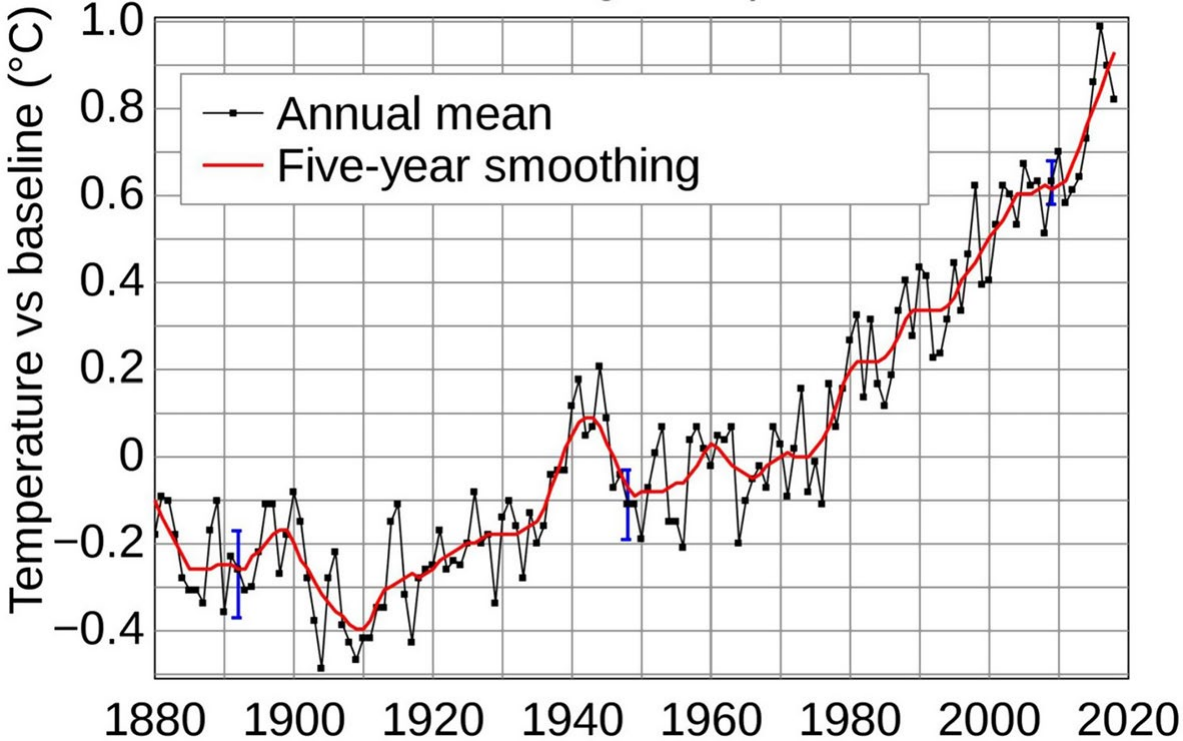
Global average temperature changes over time. Source: This figure was originally prepared by Robert A. Rohde from publicly available data and is incorporated into the Global Warming Art project

Generally, three main forces are driving these developments. The first is **global climate change**. There is no longer any doubt that global CO_2_ and other greenhouse gas emissions are progressively heating the Earth [8] (see also Fig. 1). Rising ocean temperatures produce storms of increasing intensity and duration, extending the season when tropical storms, hurricanes, typhoons, and cyclones are most frequent [9, 10]. For example, the Atlantic hurricane season stretched from August to October years ago. In the last 15 years, it expanded to June through November [11, 12].

**Fig. 2:**
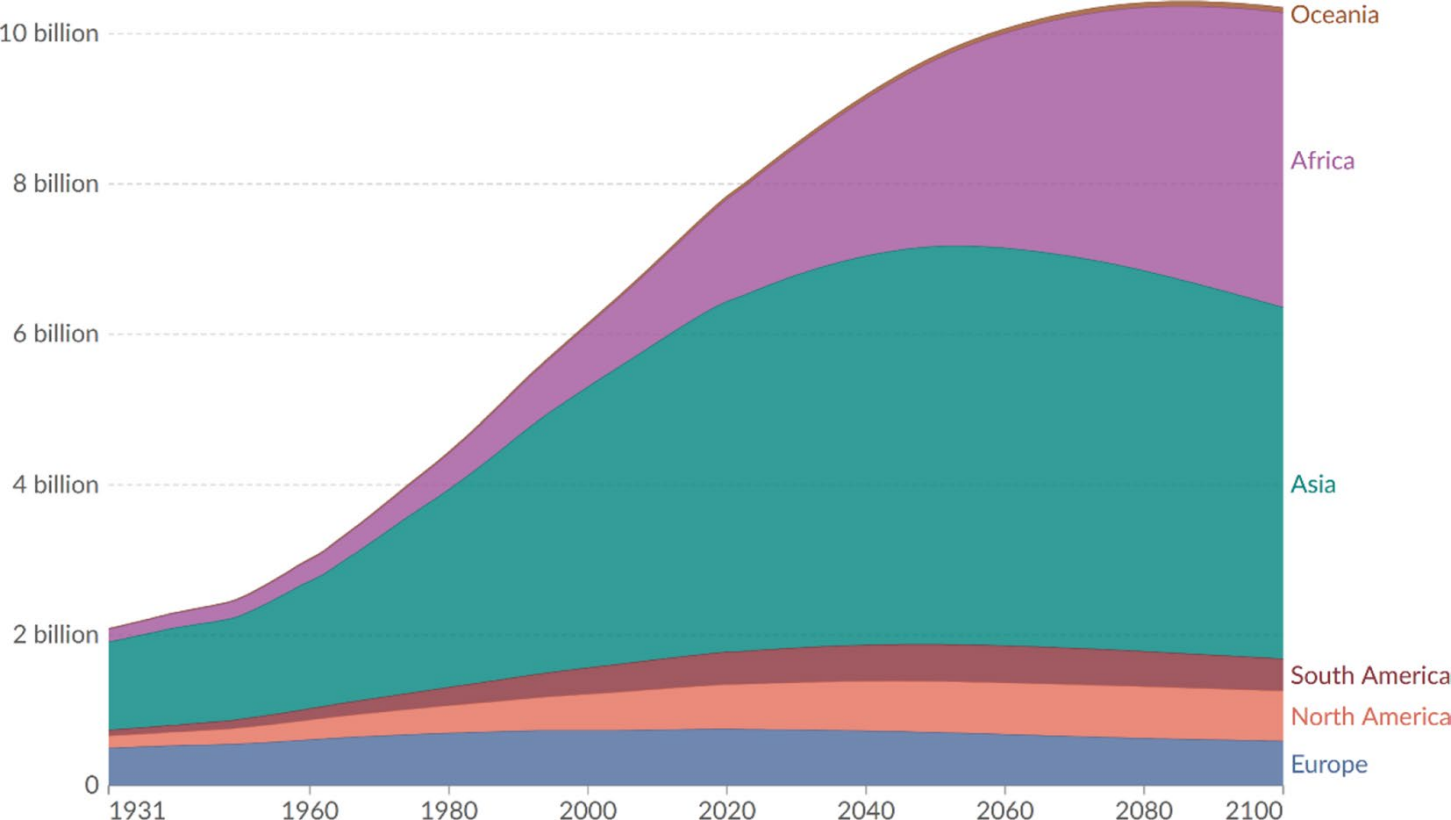
Population Growth by World Regions – Historic estimates with future projections based on the UN medium-fertility-scenario. Source: ourworldindata.org [14]

The second main driving force is **global population growth**. In 2022, the Earth’s population surpassed eight billion people. About 83 million people are added annually to the world’s population [13]. During the 20th century, the world’s population more than tripled, from 1.65 billion in 1900 to 6 billion in 1999. The United Nations projects that the world’s population could reach nearly 17 billion by 2100 [14]. See also Fig. 2.

**Fig. 3:**
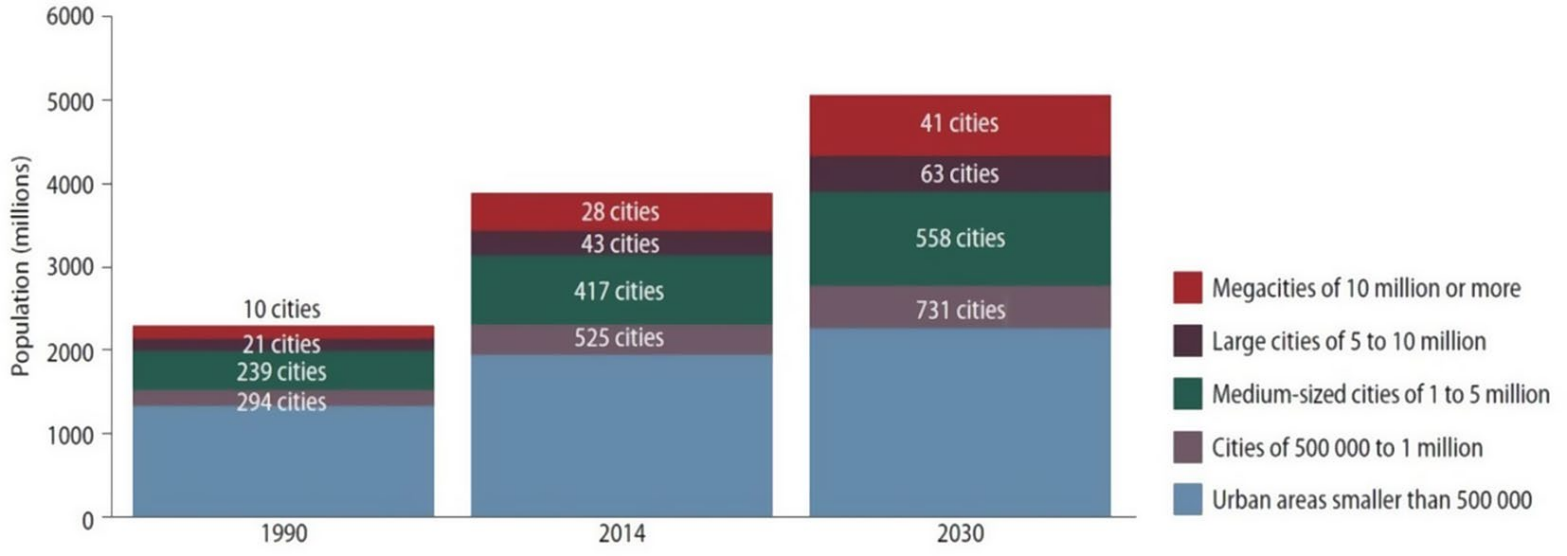
Global urban population growth is propelled by the growth of cities of all sizes. Source: UN World Urbanization Prospects [15] and Hamm et al. [16]

The third driving force is **rapid urbanization**. As the world’s population grows, more and more people are leaving rural areas and migrating to large cities in hopes of finding work and opportunity. In 1950, the percentage of people who lived in urban areas was roughly 29%. Less than 60 years later, in 2006, it was 50%. Urbanization has progressed far faster in rapidly developing countries such as China and India [15]. Within 30 years, two-thirds of the Earth’s population will live in dense urban centers [15]. See also Fig. 3. Naturally, when a disaster strikes a large city or densely populated region, the number of affected individuals may be orders of magnitude greater than those harmed in smaller-scale events.

This perspective article examines whether the current international relief and assistance paradigm should be rethought in light of the growing frequency and intensity of large-scale disasters caused by natural forces or human action.

### The principles of the current international disaster relief paradigm

**Fig. 4:**
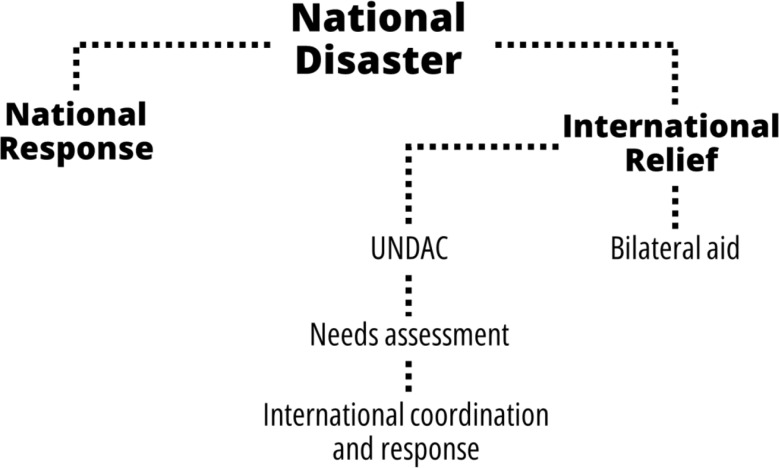
Flow chart of humanitarian relief decisions

Today, during a disaster, the affected nation (or nations), regardless of their size, decides whether it has sufficient resources to deal with the situation and assist its citizens. If it determines that it needs international assistance, it seeks aid. In this case, there are two main options. The first is to approach a country directly with which the affected country has friendly relations or contact multiple countries with bilateral agreements. The second option is to directly appeal to UN agencies that are responsible for coordinating international aid in large-scale disasters, such as the Office for the Coordination of Humanitarian Affairs (OCHA) through the United Nations Disasters Assessment and Coordination (UNDAC) and the World Health Organization (WHO) through the WHO-EMT (Emergency Medical Teams) Initiative and related agencies. In addition to these international organizations, there are large regional and global non-governmental aid organizations such as the US Agency for International Development (USAID), the International Red Cross, Physicians Without Borders (Médecins Sans Frontières), and many other aid organizations. See Fig. 4 for a flow chart of relief decisions.

In the United Nations, OCHA is responsible for coordinating humanitarian aid in the acute stages of a disaster [17]. It operates through UNDAC, a body subordinate to it that comprises people with special expertise in assessing needs and coordinating the initial response. UNDAC is largely composed of world-renowned experts in disaster management who work for UNDAC voluntarily. When a disaster-stricken country appeals to the UN, an international team of UNDAC experts is sent to the disaster within 48 h to assess the needs and help the local government coordinate international relief and take steps to mitigate its consequences. Typically, this team spends about three weeks in the country, focusing on setting immediate priorities for preventing further loss of life through search and rescue efforts, provision of necessities such as search and rescues, medical relief, food, water, clothing, shelter, and essential health, and, if possible, expediting restoration of critical infrastructure, such as transportation, energy, and communications. After about three weeks, other OCHA professionals replace the UNDAC experts and continue to assist the government with humanitarian needs and early recovery. Rapidly upon arrival, the team sends back an initial assessment of the immediate needs of the affected country that is prompted and shared with the international community through a dedicated website [17]. Countries and organizations that often offer help have continuous access to this information. Although a contribution may be monetary, many nations also send urban search and rescue (USAR) units, field clinics and hospitals (EMTs), mobile telecommunications, etc. Soon after that, clothing, tents, and equipment to restore water and food supplies to the population are usually dispatched [18, 19].

It is important to emphasize that the affected country is the sole authority to decide whether or not to request international aid and what type of aid it wants. As assets arrive, the affected country decides where they will operate and under what terms. The affected country’s government is solely responsible for meeting its citizens’ needs, from resources at its disposal or through international aid. The affected country’s government can consult with UN bodies or any other group it deems appropriate, but it retains responsibility and authority for actions taken within its borders. In parallel, potential donor countries and organizations retain the power to decide whether or not they will respond to a request for assistance, the type of assistance they will offer, and the extent and duration of their support. The goal is to meet the “golden triangle” of delivering aid in disaster situations: ensure that the right resources arrive at the right place and time. Falling short in any of these three elements diminishes the value of aid and short-changes populations in need.

History has shown that when wealthy and powerful nations deal with a major disaster, they rarely turn to the international community for assistance because they generally have sufficient resources and capabilities to manage it independently. Examples include the damage to New Orleans and the US Gulf Coast following Hurricane Katrina [20] and the Fukushima disaster in Japan [21]. If they engage other governments, it is generally with specific entities or countries to secure a special resource or expertise.

When international assistance is requested, donor countries are most inclined to help in the immediate phase. This is partially due to global attention being high and media coverage abundant. During this period, images of the disaster-affected population and their rescuers are broadcast worldwide. For various reasons, most countries prefer to send search and rescue teams, medical teams, or basic supplies such as food, medical equipment, shelters, water treatment solutions, etc., rather than monetary aid, which is less visible to the global media and the public. Once the cameras turn away and the long process of rebuilding starts, many affected countries struggle to maintain ongoing assistance [22, 23].

## Main text

### Past challenges with international humanitarian aid

If, as is widely predicted, global climate change, population growth, and rapid urbanization will increase the frequency, severity, and range of large-scale disasters, more people will be affected, the average number of casualties will increase, and the challenges faced by affected nations and the international community will be greater than those encountered in the past. The potential for several large-scale disasters to unfold simultaneously or within a short period will also increase. This will stretch the capacity of countries and international organizations to respond with the same level of resources provided in the past. For example, the budget and resources each country and relevant organization allocates to international humanitarian aid are limited. The more major disasters occur, the less funding, resources, and personnel can be allocated to each one. Alternatively, disasters occurring late in a funding cycle may get much less than those when more of the year’s budget was available.

Furthermore, throughout the world, most USAR or medical teams’ staff are volunteers. As such, they can rarely be absent from their workplace for more than a limited period. If asked to respond to several disasters per year, they may be unable to do so without harming their families or losing their jobs. Lastly, when major disasters occur close to each other in time, many donor nations are either unable or unwilling to respond with the same level of support. “Disaster fatigue” or “humanitarian fatigue” sets in [24].

Two case studies illustrate these difficulties and their challenges for future humanitarian assistance. The first case study can shed light on whether the world will have sufficient capacity to respond to several major disasters in a given year, especially if they occur over a short period. The second showcases the difficulty in creating equitable distribution of global aid.

The year 2010 was characterized by several major natural disasters: earthquakes in Haiti (January 12), Chile (February 27), and Central China (April 13), the heatwave in Russia (July to September), and severe floods in Pakistan (also July to September). These accounted for the majority of disaster-related fatalities that year (around 295,000) and just under half the overall financial losses caused by natural catastrophes [25]. The earthquake that hit Haiti on January 12, 2010, affected almost 3.5 million people, including all 2.8 million people living in the impoverished nation’s capital, Port-au-Prince. The Government of Haiti estimates that the earthquake killed 222,570 and injured another 300,572 people. Total earthquake-related financial losses were estimated at $8 billion [26]. The devastating earthquake brought an unprecedented flood of humanitarian aid [27].

The Pakistan Flood, which occurred later that same year, was one of the largest humanitarian emergencies recorded regarding the number of people affected. UN OCHA estimates indicate that nearly 2,000 people died, over 1.7 million homes were destroyed, and nearly 18 million people were affected seriously. At the worst point, approximately 20% of the nation was underwater - an area bigger than England. The country suffered extensive damage to its health, education, transportation, and communication infrastructure and crops. The total economic impact was estimated at as much as 10 billion USD. The UN initially appealed for $460 million to provide immediate aid, including food, shelter, and clean water. It later increased its request to $2 billion to support longer-term work [28].

What happened next illustrates the challenge of “disaster/humanitarian fatigue.” In August 2010, Al-Jazeera wrote: *“UN Secretary-General Ban Ki-moon had initially asked for US$460 million (€420 million) for emergency relief*, *noting that the flood was the worst disaster he had ever seen. Only 20% of the relief funds requested had been received on August 15*, *2010*” [29]. The UN voiced concerns that aid was not arriving fast enough, while the World Health Organization reported that unsafe water was consumed by ten million people left with no other choice [28]. The Pakistani economy sustained extensive damage to its infrastructure and agriculture. The total economic impact may have been as much as US$43 billion (€35 billion) [28]. Eventually, donor countries only provided an estimated 1.5 billion USD to assist in Pakistan’s recovery, less than 50% of what Haiti received seven months earlier [30]. 

Although there are likely several reasons why Haiti received a more robust response than Pakistan, including the extent of casualties, the cause of the disaster, the nation’s proximity to the US, the extent of media coverage, and the more limited geographic scope damage, perhaps the most salient point is that the Haiti earthquake happened first. By the time the floods inundated Pakistan in July-August 2010, the world’s donor countries and organizations had largely exhausted their budgets for international aid in 2010. As a result, little funding and resources were left for Pakistan. If this is the major reason Pakistan got less help, the situation will almost certainly recur as the frequency, scope, and intensity of disasters increase.

The second case study that illustrates the problematic nature of international aid allocation is the number of USAR and medical teams that responded to the earthquake in Haiti (2010) compared to those sent in response to the earthquake in Nepal (2015). In April 2015, a devastating earthquake struck Nepal. It claimed approximately 9,000 lives and injured approximately 21,000 others. Following the earthquake, 76 international USAR teams arrived in Nepal, only 18 of which were certified by The International Search and Rescue Advisory Group (INSARAG) [31]. Five years earlier, the January 2010 earthquake in Haiti, which killed approximately 220,000 people and injured more than 300,000 people, was handled by only 50 USAR teams from 30 countries, totaling 1,800 rescuers and 160 rescue dogs [32]. The disproportion is striking and speaks for itself.

Similarly, following the Nepal earthquake, 137 international emergency medical teams (EMTs) from 36 countries arrived to provide medical aid. 70% of the aforementioned medical units were NGOs, 18% were medical delegations belonging to governmental organizations, and 12% were medical units belonging to the armies of different countries [33]. Five years earlier, Medical Teams International sent about 18 volunteer teams to Haiti, the first arriving only three days after the disaster [34]. Here, too, the disproportion of the number of EMTs compared to the number of casualties in each event stands out and speaks for itself.

Following the earthquake in Haiti, a new body was established at the World Health Organization (WHO), whose role was to build an operating theory for the International dispatch of Emergency Medical Teams (EMTs), to create criteria, standards, mentoring, and a certification system, as well as to build a disaster coordination system for all medical bodies that respond to a disaster. The body did not exist in Haiti in 2010, but it was almost completely developed and activated for the second time in response to the earthquake in Nepal [35].

Sustainable aid in disaster management is crucial for long-term community resilience and empowerment. Effective disaster response requires a comprehensive approach that addresses immediate needs while fostering local capacity for ongoing rehabilitation and growth [36]. Community empowerment, involving partnership, participation, and ownership by local people, is essential for sustainable disaster management efforts [37]. This approach helps internalize disaster risk reduction tools and methods, enabling communities to better handle future risks. Integrating sustainability into humanitarian operations is challenging but necessary, requiring a shift in thinking among stakeholders, including humanitarian organizations, donors, and watchdog groups [38]. To achieve this, interdisciplinary teams must collaborate to establish immediate and long-term support networks [36]. By balancing short-term interventions with long-term sustainability goals, international relief efforts can more effectively address disaster impacts while empowering local communities for continued growth and resilience.

### Future challenges in humanitarian relief

The capabilities of the international aid community are not infinite, and in any given year, a nation’s capacity may be further constrained by economic factors, political considerations, or competing priorities. The world has a finite number of USAR teams and EMTs in any given year, and most cannot be deployed repeatedly. Sometimes, countries may tap military forces to supplement their resources, given their aptitude toward logistics, skilled personnel, and quick reaction time; however, militaries are generally less adapted to working with civilian populations and providing international humanitarian aid [39].

Many urban search and rescue teams and field hospitals rely on volunteers who are prepared to respond once a year for two to three weeks at a time to help in a disaster area. While gone, other workers must cover their duties. Most organizations support their volunteers when such deployments are infrequent, and coworkers respect their service. However, if volunteers are out two or three times a year for weeks on end, the willingness of their employers and coworkers to support their work is stretched thin. If frequent responses become the norm, this may severely curtail the number of volunteers willing to sign up.

To top it all off, the number of internationally recognized USAR teams and EMTs is limited. According to INSARAG, as of April 2024, there are a total of 126 certified USAR teams globally [40]. similarly, according to the WHO-EMT website, as of April 2024, 40 EMTs have been certified [41]. Over 50% are expanded field clinics without surgical capabilities or intensive care, defined as type 1.

Another important aspect that requires renewed thinking is the global coordination of humanitarian relief. International disaster aid mechanisms have significantly improved over the last 10–20 years. Nevertheless, work is still to be done, particularly concerning fostering greater coordination. Although UN agencies have some resources and expertise at their disposal, most major resourcing decisions are made at the national level. The type and amount of assistance each country provides may be influenced by internal political considerations, relationships between countries, and even relationships between national and international aid organizations.

As noted previously, under international law, the affected country is sovereign over which international aid missions can operate in its territory. Hence, the affected country has the authority to decide whether to accept the aid and coordinating mechanisms of the United Nations, work only with selected countries or organizations, or handle the incident independently. Furthermore, even if the affected country agrees to use the coordination mechanisms of the UN or another body, including the recommendation to accept USAR teams and/or EMTs, the affected country is not bound to follow this recommendation. It may also request and welcome rescue and medical units from other sources, including ad hoc teams of volunteers with little or no experience, who are willing to come and attempt to help. Often, these units do not bring all the required standard equipment and supplies, may lack essential personnel or expertise, and may be unable to “speak” the same professional language that UN-authorized units speak. Although these teams arrive with good intentions, their willingness to act outside guidelines and coordination mechanisms can hinder and even degrade the effectiveness of the overall response and ultimately lead to suboptimal performance.

In fact, such unofficial (non-certified) teams create a dilemma for response coordinators. If brought into the coordination mechanism, these units may augment existing teams, but they are not bound to follow the guidance they are given. Those judged as able and willing to contribute to the effort are typically welcome to participate in hopes that they will boost the response. However, incorporating units that have not been trained and certified in specific aspects of disaster response can create problems due to miscommunication or simply a lack of expertise. Moreover, fully trained and certified units may conclude that the months of years of preparation they made to achieve proficiency are not valued. If non-certified teams are welcome to join as soon as they arrive, certified teams may doubt the worthiness of expending the extra effort and cost to obtain certification.

These concerns are not theoretical. In some past disasters, host nation authorities, in concert with United Nations coordination mechanisms, have accepted the assistance of non-certified and ad hoc medical or rescue units and directed them to operate in a certain area based on needs. Occasionally, for whatever reason, these teams did not like the area of operation they were assigned to and independently moved to another location without informing the coordinating authority. In addition to confusing, unilateral actions of this sort leave affected areas short of support. When such incidents happen, the only option for the affected country is to contact the relevant government or sponsoring NGO and insist that their teams follow instructions or promptly depart the country. In practice, most host nations avoid such action to preserve international relations.

On occasion, even donor countries whose teams are part of the UN coordination mechanism decide to send different teams than those requested or establish operations in locations different than those requested by the coordinating body and the affected country’s government. Donor nations decide which units they will send, the length of time their units will stay, the equipment and supplies they will carry, and much more. Ideally, all nations would work together to optimize the mix of assistance and deploy it in such a way as to maximize its benefit. In practice, the UN coordination mechanism relies on goodwill and voluntary cooperation. The practical effect is that the coordination mechanism intended to optimize the relationship between the UN aid effort and the affected country does not work as well as it should.

On the positive side, there are several regions in the world where the UN has managed to create a higher level of coordination and commitment to cooperation with the region’s countries. This produces a more coherent and efficient response to regional disasters. Conversely, cooperative action works less effectively in other regions of the world.

The coordination mechanisms today, including the qualification and training processes of the USAR teams and EMTs, have substantially improved the quality and effectiveness of these units. The voluntary coordination mechanisms today are also preferable to the ad hoc approaches that preceded them. Although it is essential to sustain these reforms, the overarching structure to coordinate the world’s response to a single, much less multiple large-scale, disaster(s) is insufficient to meet existing needs. This is only expected to worsen with the increase in frequency and severity of global disasters.

**Steps toward a new paradigm** Although it is usually easier to diagnose the problem, it is not easy to come up with a solution. Nevertheless, in this perspective article we would like to propose several solutions.

The first is crowdsourcing of life saving capacities to the general public. For example, although internationally certified USAR units are highly trained and skilled and perform dangerous work, they are limited in their capacity to extricate alive trapped people from underneath the rubble. This is because, by the time they assemble and travel thousands of miles to reach the disaster scene, most survivors trapped alive in collapsed structures have already been rescued by family, neighbors, or local teams [42]. Those who remain trapped for more than the initial 48 h may or may not be alive by the time they are found. Numerous reports and articles from worldwide earthquakes note that 55 -95% of survivors in the first 24–48 h are rescued by non-professional laypersons [42]. Hence, one of the requested solutions, intending to increase rapid response assets with a minimum of investment, is to train citizens in earthquake-prone regions of the world with basic skills of light rescue [43, 44]. In addition to boosting the number of successful early recoveries, that approach will enable professional search and rescue units, when they arrive, to focus on sites that require more complex rescue skills. Curricula already exist to teach students in schools, youth movements, and the general public how to safely rescue family members, friends, and neighbors with the help of improvised equipment. Where there is sufficient interest, volunteer units can receive additional instruction and semi-advanced equipment [43, 44].

The second solution relies on promoting technological advancements to overcome operational gaps in relief response. For instance, in recent years, information systems have been developed to facilitate coordinated action. Artificial intelligence (AI), machine learning, and using drones and other unmanned aircraft all constitute technologies that enable a more efficient global response to disasters. Together, these advances may enhance predictive models, provide early warning of impending threats, facilitate the evacuation of coastal regions, reduce the number of casualties when disasters strike, and facilitate rapid assessment of damage in their aftermath. Similarly, information technology (IT) systems are being developed to quickly estimate the type and magnitude of response required, monitor supplies’ stocks, and optimize response efficiency.

Conversely, experience has shown that the promise of technology can be offset or even negated by its misuse by nefarious actors. Disinformation, i.e., the deliberate spread of false and misleading information through the internet, social media, and even traditional media outlets for financial or geopolitical benefit, is rampant worldwide [45]. In a disaster, it is not uncommon for cybercriminals and fraudsters to set up phony relief organizations on the Web in hopes of siphoning donations from individuals who are attempting to help victims in affected countries [46]. Finally, some nation-states conduct ongoing disinformation campaigns and actively seek to penetrate the IT systems of ministries, public utilities, corporations, NGOs of foreign adversaries, and international organizations [47]. Currently, there is a global “arms race” between those seeking to harness the benefits of information technology and those seeking to exploit its vulnerabilities for power or profit.

The third solution aims to resolve the conceptual and organizational gaps of the current international humanitarian relief paradigm. Arguably, the preferred and correct way to deal with the issue is to convene an international summit that will tackle all the objective and subjective challenges in international aid today and in the future. Such an initiative should be undertaken to bring a new paradigm into the disaster relief world, considering existing and expected problems with the full cooperation of donor countries and relief organizations, the UN agencies, and other relevant stakeholders. The call here is for convening a G-20-like summit, joined by representatives of donor countries, leading UN agencies, leading non-governmental organizations, such as the Davos World Economic Forum, and others.

The objectives of such a convention should first be to define principles for the existing international aid coordination system, which will be acceptable by all parties, preferably under the supervision of one umbrella organization (coordinating body) that all parties will widely accept. This organization will oversee the coordination of international relief efforts and subsequently have the final say in the relief efforts dispatched to an affected country. For instance, it can decide to reserve several USAR teams or EMTs for a possible future disaster to prevent the “disaster fatigue” encountered in past years. This is not an easy task, but we have no privilege to avoid giving it a try.

Second, the convention should discuss the possible enforcement mechanism that the coordinating body may utilize to tackle breaches of its decisions. If a new order is required, perhaps an enforcement mechanism is in place to maintain it. This, too, is not a simple task. Many aspects could influence the decision-making of the countries and organizations involved in international relief. For example, NGOs whose mission is international aid mostly exist from donations. A significant part of the donations comes following and during the international aid that these organizations provide. If the coordinating body decides that a specific NGO is not dispatched to a specific disaster for any reason, this decision can have a significant financial impact on the organization. In such a situation, the organization can decide whether it dispatches anyway, in breach of the coordinating body’s decision. This can undermine the effectiveness of the coordinating body in implementing a new paradigm in global disaster relief efforts.

Lastly, the convention should, at least in principle, tackle the planning and allocating the coordinating body’s annual budget and resources, including the funds pledged to disaster-stricken areas by each UN member state that commits to it. As long as this remains unfigured, the coordinating body cannot plan in advance; rather, it only acts according to post hoc decision-making. Not surprisingly, this issue is also sensitive and complex. For example, what happens when the coordinating body directs a specific donor country to donate such resources to a country affected by a disaster, but for various reasons, the donor country does not wish to contribute to this specific country?

## Conclusions

The world must prepare for larger-scale disasters based on current conditions and foreseeable developments. The future of international disaster relief depends on our ability to anticipate the challenges associated with the developing global disaster landscape and act now to strengthen cooperation, coordination, and shared responsibility among the various organizations dedicated to disaster relief. The global community must maintain a common mindset in which the international coordination mechanisms for disaster-stricken areas will be strengthened, and a stronger commitment will be pledged by donor countries, international organizations, and affected countries to coordinate global relief efforts. A holistic approach that includes building multinational agreements with large-scale commitments, strengthening coordination between countries, crowdsourcing the local response to laypersons, and strengthening the capacity and resilience of communities to take appropriate action before, during, and after a disaster will lead to a more efficient, fair and sustainable global relief system.

## Data Availability

No dataset was generated or used in this research.
